# Assessing Chronotypes by Ambulatory Circadian Monitoring

**DOI:** 10.3389/fphys.2019.01396

**Published:** 2019-11-20

**Authors:** Antonio Martinez-Nicolas, Maria Jose Martinez-Madrid, Pedro Francisco Almaida-Pagan, Maria-Angeles Bonmati-Carrion, Juan Antonio Madrid, Maria Angeles Rol

**Affiliations:** ^1^Chronobiology Lab, Department of Physiology, College of Biology, University of Murcia, Mare Nostrum Campus, IUIE, IMIB-Arrixaca, Murcia, Spain; ^2^Ciber Fragilidad y Envejecimiento Saludable (CIBERFES), Instituto de Salud Carlos III, Madrid, Spain

**Keywords:** circadian rhythm, chronotype, ambulatory circadian monitoring, light exposure, distal skin temperature, activity

## Abstract

In order to develop objective indexes for chronotype identification by means of direct measurement of circadian rhythms, 159 undergraduate students were recruited as volunteers and instructed to wear ambulatory circadian monitoring (ACM) sensors that continuously gathered information on the individual’s environmental light and temperature exposure, wrist temperature, body position, activity, and the integrated TAP (temperature, activity, and position) variable for 7 consecutive days under regular free-living conditions. Among all the proposed indexes, the night phase marker (NPM) of the TAP variable was the best suited to discriminate among chronotypes, due to its relationship with the Munich ChronoType Questionnaire (β = 0.531; *p* < 0.001). The NPM of TAP allowed subjects to be classified as early- (E-type, 20%), neither- (N-type, 60%), and late-types (L-type, 20%), each of which had its own characteristics. In terms of light exposure, while all subjects had short exposure times to bright light (>100 lux), with a daily average of 93.84 ± 5.72 min, the earlier chronotypes were exposed to brighter days and darker nights compared to the later chronotypes. Furthermore, the earlier chronotypes were associated with higher stability and day–night contrast, along with an earlier phase, which could be the cause or consequence of the light exposure habits. Overall, these data support the use of ACM for chronotype identification and for evaluation under free living conditions, using objective markers.

## Introduction

The circadian system is synchronized to the environment by exposure to robust *zeitgebers* (time givers in German), e.g., bright light during the day time and darkness at night. However, modern life and the “24-h society” have intensified exposure to artificial lighting environments both day and night, as people engagement in shift-work and leisure time is displaced toward the night-time hours. In this environment, each person shows a particular circadian preference, circadian typology, or chronotype (Di Milia et al., 2013; Wright et al., 2013).

Chronotype refers to the individual differences in activity and alertness in the morning and evening (Susman et al., 2007). Early-type (E-type) subjects naturally wake up and fall asleep earlier than late-type (L-type) individuals and therefore, they interact in a substantially different way with social timetables (Giannotti et al., 2002; Adan et al., 2012), to the extent that extreme E-types could be waking up when extreme L-types are falling asleep (Roenneberg et al., 2007). Although chronotypes are partly based on genetics (Ebisawa et al., 2001; Toh et al., 2001), they are also modulated by age (Roenneberg et al., 2007; Erren and Reiter, 2013), gender (Paine et al., 2006; Roenneberg et al., 2007; Muro et al., 2009), work schedule (Gamble et al., 2011; Juda et al., 2013; Schmidt et al., 2015), personality (Muro et al., 2009; Tonetti et al., 2009; Adan et al., 2010a, b), sun time (as opposed to local or social time) (Roenneberg et al., 2013), and light exposure (Wright et al., 2013), among other factors. On the other hand, later chronotypes are associated with greater social jetlag (SJL) (Wittmann et al., 2006) and to the uncoupling between the external time (environmental and social cues) and the internal clock, which could evolve into chronodisruption-related diseases (Erren and Reiter, 2009, 2013; Fabbian et al., 2016).

Since the 1970s, diurnal preference and chronotype have been assessed mainly through self-report questionnaires, such as the Morningness–Eveningness Questionnaire (Horne and Ostberg, 1976) and the more recent Munich ChronoType Questionnaire (Roenneberg et al., 2003). However, although extensively proven, the accuracy of such questionnaires depends on self-reporting, good recall, and the subject’s ability to complete them correctly and honestly (thus, they are less useful for cognitively impaired individuals, for example), making the detection of circadian phase in humans by understandable, objective methods a compelling need. In addition, a consensus document has very recently been published that was sponsored by the National Heart Lung and Blood Institute, the National Institute on Aging and the Sleep Research Society, entitled “Developing Biomarker Arrays Predicting Sleep and Circadian-Coupled Risks to Health.” This document establishes the need to foster the development and validation of “wearable” devices, m-Health (mobile health) applications and instruments suitable for population-based and big data approaches to create predictive models and biomarker platforms for assessing circadian health (Mullington et al., 2016; Li et al., 2017). In fact, an objective and easy-to-use method to address chronotype would allow the adaptation of shift-workers’ schedules (Erren and Reiter, 2013) or the optimization of bright light therapies (Dewan et al., 2011; Sharkey et al., 2011; Zeitzer et al., 2011). This method, combined with the development of wearable multi-sensorized devices (e.g., electronic health records) and procedures, could provide new information on an unresolved challenge, the measurement of chronodisruption, through the comparison of an individual’s internal and external times (Roenneberg et al., 2003; Erren and Reiter, 2013). This would be the key for personalized medicine, detecting unhealthy habits, measuring chronodisruption, and helping to diagnose and treat pathologies on an individual basis.

The most common marker rhythms, or rhythms used to assess the circadian system, are melatonin secretion (used as the gold standard circadian phase marker), cortisol secretion, core body temperature, and motor activity (Mormont et al., 2000; Van Someren, 2000). However, core body temperature is difficult to measure in free-living conditions (Martinez-Nicolas et al., 2017), and thus the wrist temperature rhythm has emerged as a marker rhythm, since it can be easily measured (Sarabia et al., 2008), it has been validated against polysomnography (Ortiz-Tudela et al., 2014a), and it shows a stable phase relationship with dim light melatonin onset (DLMO) (Bonmati-Carrion et al., 2014b). In the case of the motor activity rhythm, it has been clinically approved for assessing circadian rhythm disorders and several sleep pathologies (Smith et al., 2018a, b). In addition, evaluating the main *zeitgeber*—light exposure—provides information on circadian synchronization (Martinez-Nicolas et al., 2011; Wright et al., 2013) and possible phase shifts when considering the phase response curve (PRC) (Khalsa et al., 2003). However, it must be considered that the PRC depends on internal time, and thus the response to light exposure can shift according to each chronotype (Roenneberg et al., 2010). Furthermore, isolated variable monitoring rhythms can be influenced by artifacts and masking factors. Thus, it has recently been reported that multivariable recording under ambulatory conditions [ambulatory circadian monitoring (ACM)] together with composite variables can minimize these limitations (Ortiz-Tudela et al., 2010; Kolodyazhniy et al., 2011). Thus, our lab developed the integrated variable TAP, consisting of wrist temperature, motor activity, and body position (Ortiz-Tudela et al., 2010), and which has also been validated against polysomnography (Ortiz-Tudela et al., 2014a).

Therefore, the main goal of this work was to develop a method for chronotype identification based on objective markers obtained by ACM, using wearable devices that continuously gather information on wrist temperature, body position, motor activity, environmental temperature, and light exposure. The secondary objective of the work was to assess the main rhythmic characteristics of three different chronotypes (early, neither, and late), building a bridge between phase markers and chronotype classification. In this sense, our hypotheses were that ACM would allow chronotype objective identification and that different chronotypes would present different rhythmic characteristics.

## Materials and Methods

### Subjects

For this study, 159 undergraduate student volunteers (63 men and 96 women, 20.2 ± 0.4 years of age), all residents in Murcia, Spain (latitude 38°0 10°N), were recruited. Exclusion criteria were being under 18 years old, and having any physical or mental health disorder that could affect sleep (i.e., asthma, restless legs syndrome, obstructive sleep apnea, etc.), or taking any medication that could affect circadian rhythms, according to subjects’ self-reporting during a personal interview just before the ACM. None of the subjects were shift-workers or had crossed more than two time zones in the 2 months prior to study admission. All data were recorded under free-living conditions during the last week of November and the first 2 weeks of December, in order to avoid any chronotype-dependent circannual effects (Allebrandt et al., 2014). During this period of time, sunrise occurred at between 07:56 and 08:18 h, and sunset at between 17:45 and 17:50 h (data obtained from the Spanish National Geographical Institute^1^).

The study was approved by the Ethical Committee of the University of Murcia in compliance with Spanish Law (RD53/2013). Data from the volunteers were included in a database and were protected according to Spanish Law 15/1999 of 13 September. All participants received appropriate information about the study characteristics and signed an informed consent form prior to their inclusion in the study.

### Data Collection

#### Ambulatory Circadian Monitoring (ACM)

Participants were instructed to maintain their habitual lifestyle for 7 consecutive days in parallel with non-invasive circadian ambulatory monitoring of a marker rhythm (wrist temperature, WT), two variables dependent on willingness (motor activity, A, and body position, P) and two synchronizers (light exposure, L, and environmental temperature, ET) patterns, which involved wearing three sensors, as described below. If a variable was recorded for less than 7 days, it was excluded from the following analysis.

##### Wrist temperature (WT)

All subjects (*n* = 159) wore a Thermochron iButton DS1921H (Maxim Integrated Products, Sunnyvale, CA, United States) to measure WT. This temperature sensor was placed on the wrist of the non-dominant hand over the radial artery, and was isolated from the environmental temperature by a double-sided cotton sport wrist band, as previously described by Sarabia et al. (2008). The device was programmed to sample every 10 min over the course of the entire week.

##### Motor activity (A) and body position (P) rhythm measurement

The P and A rhythms were recorded every 30 s (*n* = 159), using a HOBO Pendant G Acceleration Data Logger UA-004-64 (Onset Computer, Bourne, MA, United States) placed on the non-dominant arm by means of a sport band, with its *X*-axis parallel to the humerus bone. The manufacturing specifications and the method to obtain these variables have been described in a previous work (Ortiz-Tudela et al., 2010). Briefly, motor activity was calculated as the sum of the first derivative of the angle formed between the current sensor position and its position 30 s before, considering the *X*, *Y*, and *Z* axes. The accumulated 10-min value was then calculated for comparison purposes with WT. Body position was computed as the angle between the *X*-axis of the actimeter and a horizontal plane every 30 s, and then averaged again in 10-min intervals to unify sampling frequency among the variables.

##### Light exposure (L) and environmental temperature (ET) recording

All subjects were also required to wear a HOBO Pendant Temperature/Light Data Logger UA-002-64 (Onset Computer, Bourne, MA, United States) on a neck chain to record L and ET (*n* = 137). Manufacturing specifications, memory, spectrum, and accuracy for L and ET have been previously described (Martinez-Nicolas et al., 2011, 2018). Light intensities in lux were converted into logarithmic units. L and ET were averaged in 10-min intervals to allow comparisons with WT data (Martinez-Nicolas et al., 2011, 2018).

##### Calculation of the integrated variable TAP

Wrist temperature, activity, and position (TAP) data were normalized and averaged to obtain an integrated variable (TAP) for each subject (*n* = 159). A TAP value of 0 indicates the lowest activity, a lying/horizontal position and the highest WT, which is compatible with sleep; whereas a score of 1 corresponds to periods of the highest activity, standing position and the lowest WT, which is compatible with wakefulness (see Ortiz-Tudela et al., 2010 for further details).

#### Sleep Diary

Participants were instructed to complete a sleep diary designed by the Chronobiology Lab at the University of Murcia (*n* = 148). The diary compiled information on nocturnal sleep and naps (Martinez-Nicolas et al., 2011) and sensor removals. Sleep–wake information was converted into binary values by assigning a value of 1 when the subjects declared they were asleep and 0 when they were awake, according to the sleep diary. Sleep probability (S) obtained from this binary information was individually calculated and the S values were then averaged for the entire population to calculate the percentage of individuals asleep at any given time, as previously described by Sarabia et al. (2008).

#### Chronotype Assessment

Chronotype was individually assessed as described by Roenneberg et al. (2004). MidSleep on free days corrected for sleep deficit (MSFsc) was calculated from the sleep onset for work days (Sunday to Thursday) and free days (Friday and Saturday) and the offset for work days (Monday to Friday) and free days (Saturday and Sunday) recorded in the sleep diaries and using the formula proposed by Roenneberg et al. (2004). In addition, SJL was quantified as the absolute difference between midsleep on workdays and free days (Wittmann et al., 2006).

### Data Analysis

First, data from ACM were filtered in order to eliminate erroneous measurements, such as those produced by temporarily removing the sensors (collected in the sleep diary). Second, atypical data were removed by calculating the interquartile distance (from Q1 to Q4) and then eliminating the time points with a rate of change with respect to the previous value that was higher than the interquartile distance (Marken et al., 2006). The mean waveform for all variables was calculated per individual, and then averaged per chronotype group (early, neither, and late groups). In addition, 1-week actograms were plotted per group using as threshold: (i) the third tercile values for WT and S data (33% highest values) and (ii) the first tercile values for A, P, L, ET, and TAP (33% of lowest values).

#### Non-parametrical Indexes

To analyze any possible differences among circadian patterns, we performed a non-parametrical analysis. We calculated interdaily stability (constancy of the 24-h rhythmic pattern over days, IS), intradaily variability (rhythm fragmentation, IV), and relative amplitude (rhythm amplitude, RA) (Witting et al., 1990; Van Someren et al., 1999). For those variables whose acrophase occurred during the daytime (L, ET, A, P, and TAP), RA was calculated as the difference between M10 (average for the 10 consecutive hours with the maximum values, measured in 10-min intervals) and L5 (average for the 5 consecutive hours with the minimum values, measured in 10-min intervals), divided by the sum of M10 and L5, as previously reported by Van Someren et al. (1999) for activity. However, since WT and sleep acrophases occur during the rest period, RA was calculated as the difference between M5 (average for the 5 consecutive hours with the maximum values, measured in 10-min intervals) and L10 (average for the 10 consecutive hours with the minimum values, measured in 10-min intervals), divided by their sum (Martinez-Nicolas et al., 2011). In order to normalize RA among variables, the highest and the lowest 5% values were recoded as 1 and 0, respectively, and the values in between were rescaled for the new range. The circadian function index (CFI) described by Ortiz-Tudela et al. (2010) was also estimated, but the normalized relative amplitude (NRA) was used for its calculation (instead of RA). Finally, we calculated the time spent during the morning (08:00 to 15:50 h), evening (16:00 to 23:50 h), or night (00:00 to 07:50 h) (Martinez-Nicolas et al., 2011) at different light intensities (< 10 lux or very low light, 10–100 lux or low light, 100–1000 lux or indoors bright light, and >1000 lux or outdoors bright light) for each subject (Turner and Mainster, 2008).

#### Phase Markers

The night phase markers (NPMs) were obtained using the timing for L5 (L, ET, A, P, and TAP) or M5 (WT and sleep), while the timing of L10 (WT and sleep) or M10 (L, ET, A, P, and TAP) was used as day phase markers (DPMs), as previously described (Martinez-Nicolas et al., 2011).

#### Difference From Midnight for the Night Phase Markers (DM-NPMs)

The difference from midnight for the NPMs (DM-NPMs) was intended to evaluate the synchronization to the natural light–dark cycle. To this end, the NPM was compared with the center of the natural darkness (calculated in our case from the Spanish National Geographical Institute^1^). This difference (in hours) was divided by the maximum possible difference (12 h) to obtain a value between 0 and 1. Thus, a DM-NPM value of 1 corresponds to the maximum difference (12 h), whereas a score of 0 means a complete coincidence between the phase markers and the natural darkness center, which would be the normal situation in absence of artificial light (Wright et al., 2013).

#### Circadian Health Index (CHI)

The circadian health index (CHI) integrates three main characteristics of a healthy rhythm: NRA, IS, and DM-NPM. The formula for CHI is:

$$\mathrm{CHI}=\frac{\mathrm{NRA}+\mathrm{IS}+1-\left(\mathrm{DM}-\mathrm{NPM}\right)}{3}$$

where DM-NPM was inverted to obtain values coherent with *NRA* and *IS*. Thus, *CHI* oscillates between 1 for a healthy circadian system (with high amplitude and stability, and phase coincidence of the variable NPM and the center of the natural darkness) and 0 for an altered circadian system (with low amplitude and stability, and a difference of 12 h between the NPM and the center of the natural darkness).

### Statistics

A regression analysis controlled for gender and age with a Bonferroni correction for multiple comparisons was performed to check for differences between the values from the chronotype questionnaire and NPM. A general linear model controlled for age and gender followed by a Bonferroni *post hoc* and Bonferroni correction for multiple comparisons was performed to check for differences in chronotype according to the MSFsc and non-parametric indexes for L, ET, WT, A, P, TAP, and S. Finally, a mixed effect model was applied for exposure time at different light intensities during the morning, evening, and night by ACM-based chronotypes. All statistical analyses were performed with SPSS version 23.0 (SPSS, Inc., Chicago, IL, United States). All data are expressed as mean ± SEM and were processed using Microsoft Office Excel 2016.

## Results

### Selection of Chronotypes by ACM Phase Assessment

Although among ACM-based chronotype measures, TAP and activity NPMs showed a similar strong relationship to chronotype according to the MSFsc and other phase markers (see Table 1), TAP NPM was selected due to its greater robustness and correspondence to the sleep–wake pattern (Ortiz-Tudela et al., 2010, 2014a). Three different categories were then established for this index: (i) E-type, which included those subjects within the lowest 20% for TAP NPM; (ii) L-type, for those whose NPM was located in the highest 20%; and (iii) neither type (N-type) for the remaining 60% of the subjects.

**TABLE 1:** Regression analysis among chronotype questionnaire scores and values from night phase markers for light exposure, environmental temperature, wrist temperature, activity, position, TAP, and sleep probability.

**Standardized beta**									
		**L**	**ET**	**WT**	**A**	**P**	**TAP**	**S**	**MSFsc**
	L		0.237^∗^	0.314^†^	0.760^†^	0.230^∗^	0.782†	0.748†	0.477†
	ET	137		0.051	0.107	0.043	0.154	0.220	0.318†
	WT	137	137		0.345^†^	0.090	0.431†	0.328†	0.242^∗^
	A	137	137	159		0.243^∗^	0.843†	0.806†	0.560†
n	P	137	137	159	159		0.192	0.201	0.137
	TAP	137	137	159	159	159		0.806†	0.531†
	S	127	127	148	148	148	148		0.610†
	MSFsc	127	127	148	148	148	148	148	

*Values of the upper triangular matrix are beta-standardized coefficients calculated by a regression analysis controlled for gender and age with Bonferroni correction (^∗^*p* < 0.007; †*p* < 0.001). The lower triangular matrix shows the sample size for the corresponding regression analysis. Abbreviations: L, light exposure; ET, environmental temperature; WT, wrist temperature; A, activity; P, position; TAP, integrated variable integrated by WT, A, and P; S, sleep probability; MSFsc, midsleep on free days corrected for sleep deficit (from Munich ChronoType Questionnaire).*

### Chronobiological Characterization of ACM-Based Chronotypes

The classification of the subjects according to the NPM of TAP as E- (20%), N- (60%), and L-types (20%) yielded an NPM cut-off point earlier or equal to 04:00 h for E-types, and later than 05:00 h for L-types (N-types were therefore located in between). The ACM-based chronotype classification parallels that obtained from MSFsc for E-, N-, and L-types (Table 2) with a 58.8% of coincidence between both, a 39.2% of one step errors (E- and L-types as N-types or N-types as E- or L-types) and only a 2% of E-types or L-types classified in the opposite category. However, there were no statistical differences in SJL among chronotypes (Table 2). Finally, L-types showed a delayed sleep onset and offset compared to E-types, with N-types in an intermediate position, exhibiting no differences in sleep duration (see Table 2).

**TABLE 2:** MSFsc, midsleep on free days corrected for sleep deficit, and non-parametrical indexes of the circadian rhythms (light exposure, environmental temperature, wrist temperature, activity, body position, integrated variable TAP, and sleep) according to chronotype.

**Variable**	**E (< 04:00)**	**N (04:00-05:00)**	**L (> 05:00)**	***P***
**Characteristics**				
Age	21.1 ± 0.94	20.3 ± 0.57	19.2 ± 0.12	0.206
Gender (M/W)	26/10	49/35	21/18	0.232
**MSF_*SC*_**				
*n*	34	80	34	
*h*	4.98 ± 0.21^a^	5.65 ± 0.14^b^	6.73 ± 0.21^c^	**0.000**
**SJL**				
*n*	34	80	34	
*h*	1.50 ± 0.19	1.73 ± 0.12	1.76 ± 0.19	0.525
**Sleep diary**				
*n*	34	80	34	
Sleep onset (hh:mm)	00:23 ± 00:01^a^	01:15 ± 00:01^b^	02:04 ± 00:01^c^	**0.000**
Sleep offset (hh:mm)	08:05 ± 00:01^a^	08:32 ± 00:01^b^	09:34 ± 00:01^c^	**0.000**
Sleep duration (hh:mm)	07:42 ± 00:01	07:18 ± 00:01	07:30 ± 00:01	0.034
**ET**				
*n*	33	71	32	
IS	0.47 ± 0.03	0.43 ± 0.02	0.39 ± 0.03	0.122
IV	0.12 ± 0.01	0.12 ± 0.00	0.11 ± 0.01	0.920
NRA	0.50 ± 0.05	0.38 ± 0.03	0.37 ± 0.05	0.075
NPM (hh:mm)	06:40 ± 00:40	06:12 ± 00:27	07:30 ± 00:41	0.291
DPM (hh:mm)	14:42 ± 00:37	15:56 ± 00:25	15:41 ± 00:38	0.265
L5 (^*o*^C)	18.39 ± 0.47	19.18 ± 0.32	19.38 ± 0.47	0.266
M10 (^*o*^C)	24.95 ± 0.34	25.07 ± 0.23	25.22 ± 0.34	0.846
DM-NPM	0.38 ± 0.02	0.38 ± 0.01	0.41 ± 0.02	0.346
CFI	0.64 ± 0.02	0.58 ± 0.02	0.57 ± 0.02	0.078
CHI	0.53 ± 0.02	0.48 ± 0.02	0.45 ± 0.03	0.057
**LE**				
*n*	33	71	32	
IS	0.59 ± 0.02^a^	0.51 ± 0.01^b^	0.47 ± 0.02^b^	**0.000**
IV	0.25 ± 0.01	0.25 ± 0.01	0.26 ± 0.01	0.709
NRA	0.88 ± 0.05^a^	0.78 ± 0.03^ab^	0.66 ± 0.05^b^	**0.004**
NPM (hh:mm)	04:01 ± 00:06^a^	04:40 ± 00:04^b^	05:23 ± 00:06^c^	**0.000**
DPM (hh:mm)	15:10 ± 00:17	15:36 ± 00:12	15:56 ± 00:18	0.177
L5 (log_10_lux)	0.04 ± 0.02	0.06 ± 0.01	0.10 ± 0.02	0.054
M10 (log_10_lux)	2.06 ± 0.05^a^	1.91 ± 0.04^ab^	1.79 ± 0.05^b^	**0.002**
DM-NPM	0.27 ± 0.01^a^	0.32 ± 0.01^b^	0.38 ± 0.01^c^	**0.000**
CFI	0.78 ± 0.02^a^	0.72 ± 0.01^b^	0.66 ± 0.02^c^	**0.000**
CHI	0.73 ± 0.02^a^	0.66 ± 0.01^b^	0.58 ± 0.02^c^	**0.000**
**WT**				
*n*	36	84	39	
IS	0.47 ± 0.02	0.43 ± 0.01	0.41 ± 0.02	0.100
IV	0.17 ± 0.01	0.18 ± 0.01	0.15 ± 0.01	0.114
NRA	0.47 ± 0.04	0.43 ± 0.03	0.49 ± 0.04	0.341
NPM (hh:mm)	03:22 ± 00:19^a^	03:48 ± 00:12^a^	05:35 ± 00:18^b^	**0.000**
DPM (hh:mm)	16:44 ± 00:26^a^	17:00 ± 00:17^a^	18:16 ± 00:25^b^	**0.002**
L10 (^*o*^C)	32.62 ± 0.13	32.64 ± 0.08	32.38 ± 0.12	0.185
M5 (^*o*^C)	34.86 ± 0.07	34.74 ± 0.05	34.72 ± 0.07	0.329
DM-NPM	0.21 ± 0.02^a^	0.28 ± 0.01^b^	0.37 ± 0.02^c^	**0.000**
CFI	0.62 ± 0.02	0.59 ± 0.01	0.61 ± 0.02	0.413
CHI	0.57 ± 0.02	0.52 ± 0.01	0.51 ± 0.02	0.029
**Activity**				
*n*	36	84	39	
IS	0.44 ± 0.01^a^	0.40 ± 0.01^b^	0.38 ± 0.01^b^	**0.002**
IV	0.70 ± 0.02	0.73 ± 0.01	0.70 ± 0.02	0.371
NRA	0.68 ± 0.05	0.55 ± 0.03	0.47 ± 0.05	0.008
NPM (hh:mm)	03:48 ± 00:06^a^	04:32 ± 00:04^b^	05:36 ± 00:05^c^	**0.000**
DPM (hh:mm)	17:26 ± 00:17	17:19 ± 00:11	18:10 ± 00:16	0.032
L5 (^*o*^/min)	14.92 ± 0.86^a^	16.88 ± 0.56^ab^	19.16 ± 0.82^b^	**0.002**
M10 (^*o*^/min)	74.24 ± 1.70	73.60 ± 1.12	76.94 ± 1.64	0.238
DM-NPM	0.25 ± 0.01^a^	0.31 ± 0.01^b^	0.40 ± 0.01^c^	**0.000**
CFI	0.59 ± 0.02	0.53 ± 0.01	0.50 ± 0.02	0.008
CHI	0.62 ± 0.02^a^	0.55 ± 0.01^b^	0.48 ± 0.02^c^	**0.000**
**Position**				
*n*	36	84	39	
IS	0.54 ± 0.02	0.52 ± 0.02	0.45 ± 0.02	0.018
IV	0.28 ± 0.02	0.28 ± 0.01	0.30 ± 0.02	0.781
NRA	0.65 ± 0.05	0.65 ± 0.03	0.58 ± 0.04	0.443
NPM (hh:mm)	04:47 ± 00:24	04:53 ± 00:16	06:18 ± 00:23	0.006
DPM (hh:mm)	15:05 ± 00:27	16:18 ± 00:18	16:42 ± 00:26	0.028
L5 (^*o*^)	16.37 ± 0.81	16.95 ± 0.53	17.99 ± 0.77	0.336
M10 (^*o*^)	52.21 ± 1.11	53.29 ± 0.73	51.69 ± 1.07	0.421
DM-NPM	0.28 ± 0.01^a^	0.32 ± 0.01^b^	0.39 ± 0.01^c^	**0.000**
CFI	0.68 ± 0.02	0.67 ± 0.02	0.63 ± 0.02	0.199
CHI	0.64 ± 0.02	0.61 ± 0.02	0.55 ± 0.02	0.013
**TAP**				
*n*	36	84	39	
IS	0.62 ± 0.02^a^	0.57 ± 0.01^ab^	0.53 ± 0.02^b^	**0.002**
IV	0.24 ± 0.01	0.25 ± 0.01	0.23 ± 0.01	0.596
NRA	0.66 ± 0.04	0.58 ± 0.03	0.54 ± 0.04	0.141
NPM (hh:mm)	03:34 ± 00:04^a^	04:36 ± 00:03^b^	05:45 ± 00:04^c^	**0.000**
DPM (hh:mm)	17:15 ± 00:15^a^	16:57 ± 00:10^a^	18:06 ± 00:14^b^	**0.000**
L5 (a. u.)	0.15 ± 0.01	0.17 ± 0.01	0.17 ± 0.01	0.262
M10 (a. u.)	0.63 ± 0.01	0.62 ± 0.00	0.61 ± 0.01	0.488
DM-NPM	0.23 ± 0.01^a^	0.31 ± 0.00^b^	0.41 ± 0.01^c^	**0.000**
CFI	0.72 ± 0.02	0.68 ± 0.01	0.65 ± 0.02	0.070
CHI	0.68 ± 0.02^a^	0.61 ± 0.01^b^	0.55 ± 0.02^c^	**0.000**
**Sleep**				
*n*	34	81	34	
IS	0.76 ± 0.02^a^	0.69 ± 0.01^b^	0.61 ± 0.02^c^	**0.000**
IV	0.08 ± 0.00	0.09 ± 0.00	0.08 ± 0.00	0.080
NRA	1.00 ± 0.05	0.84 ± 0.03	0.89 ± 0.05	0.026
NPM (hh:mm)	04:07 ± 00:05^a^	04:36 ± 00:04^b^	05:38 ± 00:05^c^	**0.000**
DPM (hh:mm)	16:47 ± 00:11^a^	17:17 ± 00:08^a^	18:22 ± 00:12^b^	**0.000**
L10 (%)	2.06 ± 0.51	3.32 ± 0.33	3.31 ± 0.51	0.097
M5 (%)	94.97 ± 1.48^a^	92.80 ± 0.96^a^	88.51 ± 1.48^b^	0.008
DM-NPM	0.27 ± 0.01^a^	0.31 ± 0.00^b^	0.40 ± 0.01^c^	**0.000**
CFI	0.91 ± 0.02^a^	0.83 ± 0.01^b^	0.82 ± 0.02^b^	**0.002**
CHI	0.60 ± 0.02^a^	0.54 ± 0.01^b^	0.52 ± 0.02^b^	**0.001**

*Values are expressed as mean ± SEM. *p* denotes global significance level according to general linear model controlled for age and gender (in bold probability values lower than 0.005, as indicated by Bonferroni’s correction for multiple comparisons). Different superscript letters denote significant differences among chronotypes for each variable and index. Abbreviations: M, men; W, women; E, early-type; N, neither-type; L, late-type; MSFsc, midsleep on free days corrected for sleep deficit; SJL, social jetlag; LE, light exposure; ET, environmental temperature; WT, wrist temperature; IS, interdaily stability; IV, intradaily variability; NRA, normalized relative amplitude; L5, mean of the 5 consecutive hours with the lowest values; M10, mean of the 10 consecutive hours with the highest values; M5, mean of the 5 consecutive hours with the highest values; L10, mean of the 10 consecutive hours with the lowest values; NPM, night phase marker; DPM, day phase marker; DM-NPM, difference from midnight for the night phase markers; CFI, circadian function index; CHI, circadian health index.*

**FIGURE 1:**
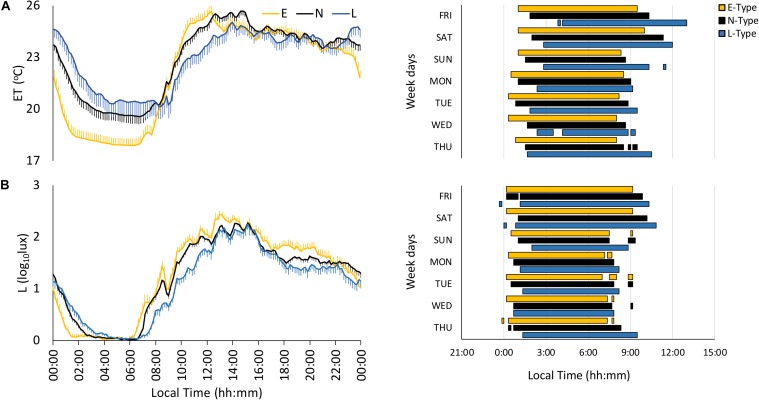
Mean waveforms and actograms for early (E-types, in yellow; *n* = 20% of total subjects), neither (N-types, in dotted black; *n* = 60% of total subjects), and late-types (L-types, in blue; *n* = 20% of total subjects) for: **(A)** light exposure (L; total subjects = 137) and **(B)** environmental temperature (ET; total subjects = 137). Waveform data are expressed as mean ± SEM. Plotted actograms correspond to the first tercile values (values corresponding to the rest phase).

The environmental temperature (ET) pattern presented low values at night and high values during the daytime (Figure 1A). E-types tended to exhibit greater stability (IS), amplitude (NRA), robustness (CFI), and CHI, along with an earlier NPM, than L-types (Figure 1A), and N-types presented intermediate values, as shown in Figure 1A and Table 2; however, these differences were not statistically significant in any case (Table 2).

The light exposure pattern exhibited low and stable values at night and higher and variable values during daytime, with clear variations in phase and light intensity among chronotypes (Figure 1B). As expected, E-types showed an earlier light offset and onset than L-types, with the N-types in an intermediate position for both work and free days (see Figure 1B). In addition, E-types showed higher stability (IS), amplitude (NRA), daytime values (M10), robustness (CFI), and CHI, and lower difference between midnight (DM-NPM) and NPMs than L-types. Once again, N-types showed an intermediate position that differed significantly from E-types in terms of IS and from the other two chronotypes with regard to CFI, CHI, DM-NPM, and NPM (Table 2). In addition, and according to the light intensity analysis (Table 3), the subjects were generally exposed to bright light (>1000 lux) for a mean duration of 93.84 ± 5.72 min. E-types spent less time at very low light levels (< 10 lux) during the morning and evening, at the expense of increasing their exposure time to indoor bright light (100–1000 lux) or even outdoor bright light (>1000 lux). L-types show the opposite behavior, with the lowest exposure time to outdoor lighting during the day, spending most of their diurnal time in the lowest light categories. As expected, N-types exhibit intermediate situations.

**TABLE 3:** Duration and intensity of light exposure by chronotype and daytime interval.

**Daytime interval**	**Chronotype**	**< 10 lux**	**10–100 lux**	**100–1000 lux**	**>1000 lux**
Morning 08:00–15:50	E-type	1:23 ± 0:10^a^	2:06 ± 0:08^a^	3:05 ± 0:13^b^	1:25 ± 0:14^*a*^
	N-type	2:07 ± 0:08†^*a*^	2:17 ± 0:07^ab^	2:42 ± 0:08^b^	0:54 ± 0:05^c^
	L-type	2:56 ± 0:19†‡^a^	1:56 ± 0:11^b^	2:19 ± 0:13†^ab^	0:49 ± 0:05^c^
Evening 16:00–23:50	E-type	2:18 ± 0:17^∗^^ab^	2:01 ± 0:10^a^	3:05 ± 0:24^b^	0:37 ± 0:16^∗^^c^
	N-type	2:09 ± 0:09^a^	2:46 ± 0:09†^∗^^a^	2:41 ± 0:11^a^	0:24 ± 0:05^∗^^b^
	L-type	3:25 ± 0:23†‡^a^	2:14 ± 0:14^b^	2:08 ± 0:17†^b^	0:13 ± 0:03^∗^^c^
Night 00:00–07:50	E-type	7:25 ± 0:05^∗^#^a^	0:13 ± 0:02^∗^#^b^	0:20 ± 0:04^∗^#^b^	0:02 ± 0:01^∗^#^b^
	N-type	6:56 ± 0:06†^∗^#^a^	0:28 ± 0:04^∗^#^b^	0:32 ± 0:04^∗^#^b^	0:04 ± 0:01^∗^^*c*^
	L-type	6:51 ± 0:10†^∗^#^a^	0:38 ± 0:05†^∗^#^b^	0:29 ± 0:06^∗^#^b^	0:01 ± 0:01^∗^^c^

*Duration and intensity of light exposure during morning, evening and night by chronotype for all the subjects (*n* = 137). Values are expressed as mean ± SEM (h:min). † Indicates differences with E-type for the same light intensity and daytime interval, ‡ indicates differences with N-type in the same light intensity and daytime interval. ^∗^ Indicates differences with Morning for the same light intensity and chronotype, # indicates differences with Evening for the same light intensity and chronotype. Different letters denote differences among light intensities for the same daytime interval and chronotype. Duration and intensity of light exposure during morning, evening and night by chronotype for all the subjects (*n* = 137). Values are expressed as mean ± SEM (h:min). † indicates differences with E-type for the same light intensity and daytime interval, ‡ indicates differences with N-type in the same light intensity and daytime interval, ^∗^ indicates differences with morning for the same light intensity and chronotype, and # indicates differences with evening for the same light intensity and chronotype. Different letters denote differences among light intensities for the same daytime interval and chronotype.*

**FIGURE 2:**
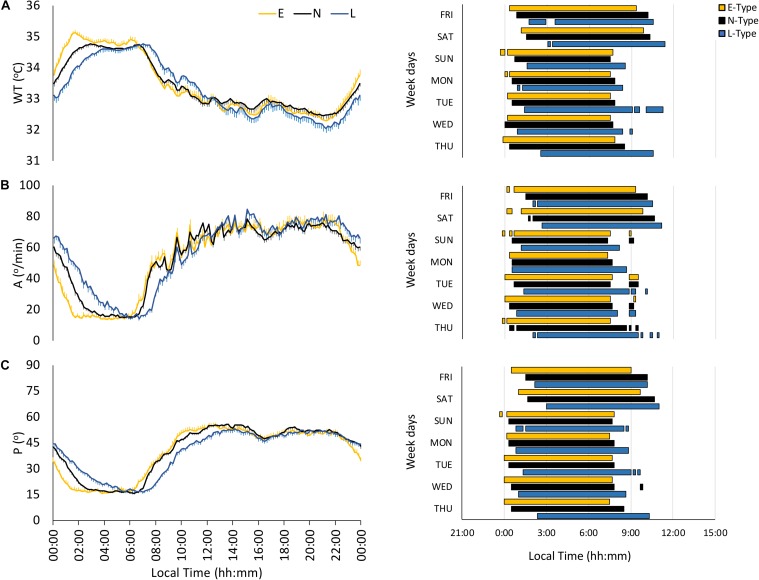
Mean waveforms and actograms for early (E-types, in yellow; *n* = 20% of total subjects), neither (N-types, in dotted black; *n* = 60% of total subjects), and late-types (L-types, in blue; *n* = 20% of total subjects) for: **(A)** wrist temperature (WT; total subjects = 159), **(B)** activity (A; total subjects = 159), and **(C)** body position (P; total subjects = 159). Waveform data are expressed as mean ± SEM. WT actogram corresponds to the third tercile values, while activity and body position actograms correspond to the first tercile values (values corresponding to the rest phase).

The wrist temperature (WT) pattern presented a plateau of high values at night and low values during daytime, with E-types phase advanced as compared to L-types (Figure 2A and Table 2). However, N-types showed an intermediate WT pattern, increasing at night between E- and L-types and decreasing in the morning, similarly to E-types (see Figure 2A). Finally, E-types and N-types showed higher earlier DPM and NPM than L-types (Table 2). In addition, DM-NPM showed significant differences among the three chronotypes, with L-types having the highest values and E-types having the lowest values (Table 2).

The motor activity pattern exhibited low values at night and high values during daytime (Figure 2B), similar to those previously described, with an earlier phase for E- and N-types and a gradated nocturnal decrease according to each chronotype (Table 2). In fact, N-types presented an E-type-like behavior on workdays and an intermediate behavior on free days (see Figure 2B and Table 2). In addition, E-types showed higher stability (IS) and CHI, and lower night values (L5) and DM-NPM, along with a phase advance for NPM compared to L-types (Table 2).

As expected, the body position pattern showed low values (horizontal positions) at night and high values (vertical positions) during the daytime, with E-types showing a lower DM-NPM and an earlier phase marginally significant for NPM, compared to that of L-types (Figure 2C and Table 2), and N-types again exhibited an intermediate behavior throughout the day (Figure 2C), which is also reflected in their circadian parameters (Table 2).

**FIGURE 3:**
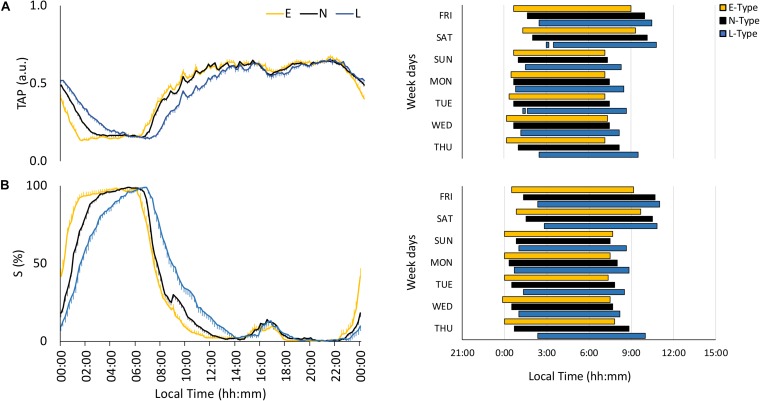
Mean waveforms and actograms for early (E-types, in yellow; *n* = 20% of the total subjects), neither (N-types, in dotted black; *n* = 60% of the total subjects), and late-types (L-types, in blue; *n* = 20% of the total subjects) for: **(A)** TAP (total subjects = 159) and **(B)** sleep (S; total subjects = 148). Waveform data are expressed as mean ± SEM. TAP and sleep actograms correspond to the first and third tercile values, respectively (values corresponding to the rest phase).

The integrated variable TAP showed low values at night and high values during the daytime, with clear phase variations among chronotypes, especially on free days (Figure 3A). E-types exhibited higher stability (IS), circadian health (CHI), advanced NPM and DPM, respectively, and lower differences from midnight on for NPM compared to L-types (Table 2). Furthermore, N-types showed an intermediate behavior compared to the other two chronotypes (Table 2).

As expected, the sleep probability pattern, calculated based on sleep diaries, was only high during the night, with a phase shift according to the chronotype (Figure 3B); this shift was present during the entire week at sleep onset, whereas N-types had an E-type sleep offset on working days and an intermediate position on free days (Figure 3B). Circadian parameters per chronotype were consistent with this behavior (Table 2).

Finally, actograms (right part of Figures 1–3) that allow SJL to be inferred by comparing work day and weekend sleep periods showed that the phase delay of L-types was more pronounced on the weekends.

## Discussion

Our results support the use of the TAP NPM obtained from ACM as an objective instrument to assess chronotype. Therefore, and as expected, eveningness was associated with a phase delay, which was more pronounced during free days, and with a less robust rhythmicity, as reflected by their lower amplitude and stability. However, we did not find any association between chronotype and SJL, which could be due to the homogeneity and youth of our population (a possible limitation of this study). Furthermore, the lifestyle of the later types was associated to darker days and brighter nights.

Although the sleep parameters obtained from the activity rhythm (sleep onset and offset) have been proposed recently as a measurement of ACM-based chronotypes (Santisteban et al., 2018), according to our results, the NPM of TAP and activity variables showed regression results similar to the MSFsc, according to the Munich ChronoType Questionnaire. This fact, along with TAP correspondence to the sleep pattern (Ortiz-Tudela et al., 2010, 2014a) and DLMO (Bonmati-Carrion et al., 2014a, b), makes the NPM of the integrated variable TAP a more suitable chronotype measurement. Wrist temperature (WT), one of the components of the TAP variable, is a marker of peripheral vasodilation mediated by the sympathetic/parasympathetic balance (Rubinstein and Sessler, 1990; Charkoudian, 2003; Sarabia et al., 2008), with a stable phase relationship with DLMO (Bonmati-Carrion et al., 2014b) and core body temperature (Kräuchi and Wirz-Justice, 1994). However, the strong endogenous component of WT would be less sensitive to social time and other external variables influencing chronotype, whereas activity and body position, also integrated in the TAP variable (Ortiz-Tudela et al., 2010), would improve the assessment.

Although other integrative methods have been reported, compiling information from several variables (core body, skin, and environmental temperatures, light exposure activity, position, etc.), they require complicated mathematical oscillator models (Jewett et al., 1999; St Hilaire et al., 2007a, b), multiple regression modeling (Kolodyazhniy et al., 2011), or a neural network (Kolodyazhniy et al., 2012) for phase assessment. The complexity of the oscillator models, the intricacy of the outcome, which complicates the multiple regression model interpretation, and, in the case of the neural network the fact that it is impossible to know how the results are obtained, all make the application of these models difficult. The TAP variable simultaneously considers the circadian endogenous component of WT and those more dependent on willingness, such as activity or body position (Ortiz-Tudela et al., 2010) in order to obtain an intelligible and comprehensive assessment of the subject’s circadian preferences, while at the same time considering most of the factors that influence this preference. Although beyond the scope of the present study, it could be interesting in future research to analyze sleep parameters in more detail using TAP variable (Ortiz-Tudela et al., 2014a) or new ACM devices as those recently described (Madrid-Navarro et al., 2019).

The pattern of ACM variables has been previously described (Sarabia et al., 2008; Ortiz-Tudela et al., 2010; Martinez-Nicolas et al., 2011, 2014) and as expected, in our study those variables with a nocturnal acrophase exhibited stable values at night and greater variability during the daytime, while the opposite occurred with those variables that have their acrophase during the daytime. As opposed to L-types, E-types showed more stable rhythms for light exposure, activity, TAP, and sleep, which has been related to a better health status (Zuurbier et al., 2015). This is consistent with previous studies describing E-types as being stricter in their schedules than L-types (Adan et al., 2012). Morningness also seems to be associated with greater relative amplitude for light exposure (due to higher daytime values), activity (lower nighttime values), and better circadian health (CHI), conditions which have previously been related to a young circadian system (Martinez-Nicolas et al., 2014, 2018).

Besides in this work, eveningness has been related to a worse CFI, the low values of which have been previously associated with pathological states, such as sleep apnea (Martinez-Nicolas et al., 2017) and even cancer (Ortiz-Tudela et al., 2016). However, this index addressed day to day stability, day–night differences, and pattern fragmentation, excluding information about the circadian phase (Ortiz-Tudela et al., 2010). In order to also consider synchronization, we therefore propose the CHI, which is based on three circadian parameters: (i) interdaily stability (Witting et al., 1990), as an index of the stability among days; (ii) NRA (Van Someren et al., 1999; Martinez-Nicolas et al., 2011), as a measure of day–night contrast; and (iii) the DM-NPMs, as a measure of synchronization to the natural light–dark cycle (Wright et al., 2013). E-types seemed more synchronized with the natural light-dark cycle, as already suggested (Wright et al., 2013), since they showed lower differences from midnight for most NPM indexes than L-type individuals (except for ET).

The light exposure analysis reflected that a large proportion of the subjects spent most of their time at low light intensities (probably indoors), with brief exposure to bright light outdoors (>1000 lux), as previously described for young adults (Savides et al., 1986; Espiritu et al., 1994; Hubert et al., 1998; Mishima et al., 2001; Martinez-Nicolas et al., 2011), which results in a low day–night contrast (Martinez-Nicolas et al., 2014). This short time of bright light exposure facilitates circadian disruption as a result of the mismatch between internal and external time (Martinez-Nicolas et al., 2014). According to the chronotype, E-types presented the best exposure pattern, although they were still exposed mostly to indoor bright light. L-types spent most of their daytime underexposed to bright light, while their time in darkness during the night was shorter than for E-types, as has been previously published (Carissimi et al., 2016), probably due to the use of electronic devices (Cain and Gradisar, 2010; Chang et al., 2014; Touitou et al., 2016) until late at night in the L-type group. However, it is still unclear whether differences in voluntary light exposure between chronotypes, which could be explained by the sleep/wake pattern, are the cause or consequence of their circadian phase preference, although differences between them are minimized when both are exposed to natural light–dark cycles (Wright et al., 2013). In this sense, the possible seasonal effect similar to that already reported for MSFsc (Kantermann et al., 2007) should be evaluated in further studies.

Simultaneously assessing the circadian phase of multiple individual variables over long periods makes it possible to evaluate circadian sleep disorders, SJL, and chronodisruption (generated by the abnormal coupling of internal and external time), in order to apply countermeasures, such as those already proposed (Martinez-Nicolas et al., 2014). Many of these measures have been shown to be effective. For example, they have been successfully used to personalize chronotherapy in cancer patients (Lévi and Okyar, 2011), optimize the treatment and effectiveness of bright light therapy (Dewan et al., 2011;Sharkey et al., 2011; Zeitzer et al., 2011), and match shift-workers’ timetables to their chronotype (Erren and Reiter, 2013). These measures have also been effective in adjusting school starting times and rearranging schedules, in an effort to improve school performance (Zerbini and Merrow, 2017). It could even be possible to reduce SJL (Zerbini et al., 2018), by prompting subjects to diminish blue light at night, while increasing bright light during the morning. However, this methodology, which could be useful in the future, should be tested for shift-workers and in bright light therapy optimization.

Furthermore, only multivariable recording would provide a reliable method to assess, not only SJL, but also chronodisruption. ACM recording lasts a week (at least), and thus data gathered under free-living conditions represent a “week-type” that does not rely on our recall, as occurs with subjective tests, which can be biased (Bonmati-Carrion et al., 2014a). In addition, this method is applicable in any situation, even if the subjects cannot complete a questionnaire. In this sense, the method is also feasible in a wide variety of populations such as babies (Batinga et al., 2015), elderly subjects (Batinga et al., 2015; Martinez-Nicolas et al., 2018), Parkinson’s disease patients (Madrid-Navarro et al., 2018), patients with mild cognitive impairments (Ortiz-Tudela et al., 2014b), sleep disordered breathers (Martinez-Nicolas et al., 2017), cancer patients (Ortiz-Tudela et al., 2016), and shift workers (Gomez-Garcia et al., 2016), to mention some examples in which the patients wore the sensors without reporting any problems or side effects. In fact, most missing data occurred when sensors were removed for personal hygiene and the subjects forgot to put them back on, or took a shower with them on. Furthermore, it is important to bear in mind that measuring chronodisruption, either understood as an internal–external desynchronization (Erren and Reiter, 2013) or internal order impairment (Bonmati-Carrion et al., 2014a) requires multiple recordings of both input and output circadian signals, which is made possible thanks to ACM. Summarizing, ACM represents an objective, ambulatory, and non-invasive method that allows us to objectively assess an individual’s chronotype, and their SJL, under free-living conditions by means of recording multiple variables for internal (endogenous chronotype) and external times (solar and social times).

## Data Availability Statement

The raw data supporting the conclusions of this manuscript are available on request to the corresponding author.

## Ethics Statement

The studies involving human participants were reviewed and approved by the Ethical Committee of the University of Murcia. The patients/participants provided their written informed consent to participate in this study.

## Author Contributions

AM-N, MM-M, JM, and MR had the initial conception of the study. AM-N and MM-M performed the data analysis. AM-N, JM, and MR recruited and monitored subjects. AM-N, PA-P, JM, and MR wrote the main manuscript text, which was then discussed and refined with the other authors (AM-N, MM-M, PA-P, M-AB-C, JM, MR).

## Conflict of Interest

The authors declare that the research was conducted in the absence of any commercial or financial relationships that could be construed as a potential conflict of interest.

## Footnotes

1 http://astronomia.ign.es
